# Biphenyl-Based Covalent Triazine Framework/Matrimid^®^ Mixed-Matrix Membranes for CO_2_/CH_4_ Separation

**DOI:** 10.3390/membranes11100795

**Published:** 2021-10-19

**Authors:** Stefanie Bügel, Quang-Dien Hoang, Alex Spieß, Yangyang Sun, Shanghua Xing, Christoph Janiak

**Affiliations:** 1 Institut für Anorganische Chemie und Strukturchemie, Heinrich-Heine-Universität Düsseldorf, D-40204 Düsseldorf, Germany; stefanie.buegel@uni-duesseldorf.de (S.B.); quang-dien.hoang@uni-duesseldorf.de (Q.-D.H.); alex.spiess@hhu.de (A.S.); yasun100@hhu.de (Y.S.); shanghua.xing@hhu.de (S.X.); 2 Hoffmann Institute of Advanced Materials, Shenzhen Polytechnic, 7098 Liuxian Blvd, Nanshan District, Shenzhen 518055, China

**Keywords:** mixed-matrix membrane (MMM), covalent triazine framework (CTF), Matrimid^®^, CO_2_/CH_4_ separation

## Abstract

Processes, such as biogas upgrading and natural gas sweetening, make CO_2_/CH_4_ separation an environmentally relevant and current topic. One way to overcome this separation issue is the application of membranes. An increase in separation efficiency can be achieved by applying mixed-matrix membranes, in which filler materials are introduced into polymer matrices. In this work, we report the covalent triazine framework CTF-biphenyl as filler material in a matrix of the glassy polyimide Matrimid^®^. MMMs with 8, 16, and 24 wt% of the filler material are applied for CO_2_/CH_4_ mixed-gas separation measurements. With a CTF-biphenyl loading of only 16 wt%, the CO_2_ permeability is more than doubled compared to the pure polymer membrane, while maintaining the high CO_2_/CH_4_ selectivity of Matrimid^®^.

## 1. Introduction

Since the first large-scale application of a hydrogen-separating membrane in the 1980s [1], a strong development in membrane technology for various separation tasks has been noticeable. One of these tasks is the separation of CO_2_ and CH_4_ (for “biogas upgrading” and “natural gas sweetening”), for which the use of polymeric membranes is an appropriate option, since they offer an energy- and cost-efficient separation [2,3]. In order to further increase the CO_2_ permeability and CO_2_/CH_4_ selectivity of gas separation membranes, additional filler components are used as a dispersed phase embedded in a polymer matrix to form mixed-matrix membranes (MMMs) [4]. In addition to frequently utilized filler materials, such as metal-organic frameworks (MOFs) [5,6,7,8,9,10], including zeolitic imidazolate frameworks (ZIFs) [11,12,13] or zeolites [14,15], purely organic porous materials, such as covalent organic frameworks (COFs) or covalent triazine frameworks (CTFs), are also promising materials [16,17]. Especially, in a completely organic MMM, a good compatibility of filler and matrix is more likely than in MMMs with inorganic fillers so that the formation of the unselective void volume may be decreased [18]. Shan et al. synthesized the microporous azine-linked COF material ACOF-1 and incorporated it into a Matrimid^®^ matrix. The gas separation performance was tested with an equimolar CO_2_/CH_4_ mixture at 308 K, and the 16 wt% MMM showed a rise in CO_2_ permeability of 8.5 Barrer without loss of CO_2_/CH_4_ selectivity [19]. The fluorinated CTF FCTF-1 was applied as filler material in a PIM-1 membrane. The CO_2_ permeability increased from 5800 Barrer for the pure membrane to 9400 Barrer for the 5 wt% FCTF-1 membrane and the CO_2_/CH_4_ selectivity was improved from 11.5 to 14.8 [20]. Previous studies showed that the incorporation of CTF-1 in a PSF matrix exhibited an elevation in CO_2_ permeability from 7.3 Barrer for the pristine membrane to 12.7 Barrer for a filler content of 24 wt% CTF-1 whilst maintaining the CO_2_/CH_4_ selectivity [21]. 

This ability of organic filler materials to enhance the performance of MMMs was taken as a reason to prepare novel CTF-biphenyl/Matrimid^®^ MMMs. The glassy polymer Matrimid^®^ was chosen as a matrix due to its high CO_2_/CH_4_ selectivity, thermal stability [22], and the previously proven good compatibility with CTF materials [23]. Further, studies on the effect of the ionic liquid (IL) 1-butyl-3-methylimidazolium bis(trifluoromethanesulfonyl)imide, [BMIm][NTf_2_] on membrane performance were investigated due to the high solubility of CO_2_ in ILs [24,25,26] and the positive effects of ILs on membrane performance, which were reported previously [27,28,29,30]. CO_2_ and CH_4_ mixed-gas permeabilities and selectivities were measured for all membrane systems. 

## 2. Materials and Methods

### 2.1. Materials

For the CTF-biphenyl synthesis, anhydrous aluminum chloride (AlCl_3_; 98.5%) was received from Arcos Organics, cyanuric chloride (99%) was obtained from Sigma-Aldrich, and biphenyl (99%) from Alfa Aesar. Biphenyl was recrystallized from ethanol before usage. For the synthesis of the IL, [BMIm][NTf_2_] 1-chlorobutane (99%) was purchased from Merck, 1-methylimidazole (99%) and lithium bis(trifluoromethanesulfonyl)imide (Li[NTf_2_]; 99%) were obtained from Fluorochem, activated charcoal (extra pure) from Merck, and acidic aluminum oxide (150 m^2^/g; Brockmann grade 1) from Alfa Aesar. The solvents acetone (≥99.8%), dichloromethane (DCM; 99.99%), and ethyl acetate (≥99%) were received from Fisher Scientific; and methanol (MeOH; ≥99.8%) and tetrahydrofuran (THF; ≥99.9%) from Sigma-Aldrich. The polymer Matrimid^®^ 5218 (BTDA/DAPI) was provided by Huntsman Advanced Materials. The gases CO_2_ (grade 4.5), CH_4_ (grade 4.5), and He (grade 5.0) were received from Air Liquide.

### 2.2. Synthesis of CTF-Biphenyl 

**Scheme 1:**
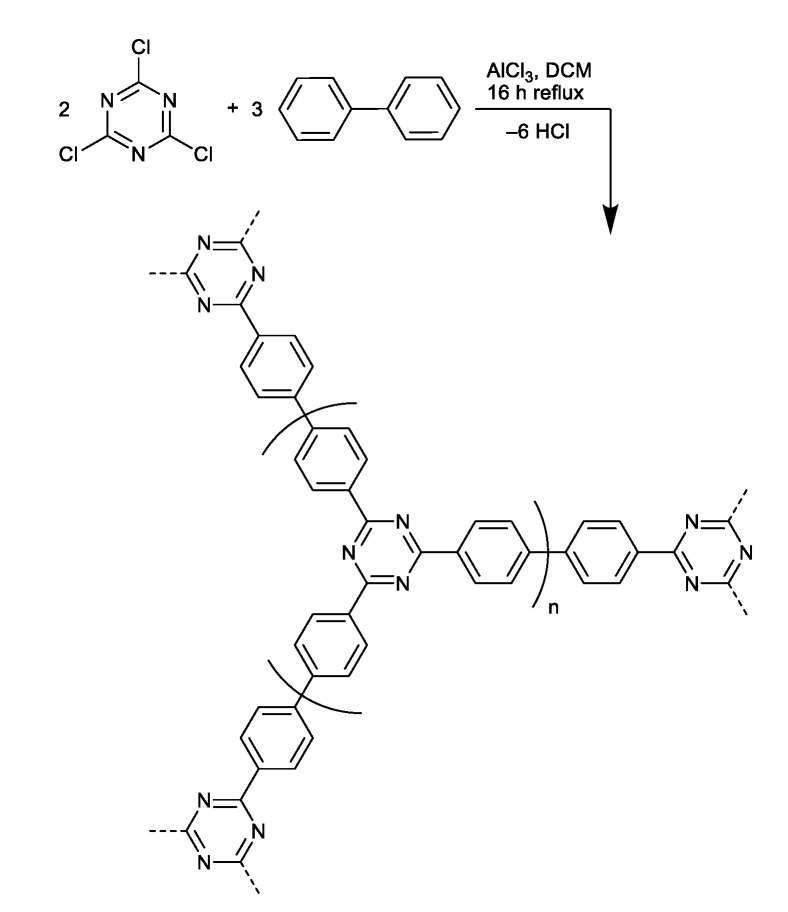
Synthesis of CTF-biphenyl (idealized structure with repeat unit in parenthesis).

CTF-biphenyl was synthesized by Friedel-Crafts alkylation (Scheme 1) due to the possible upscaling, which is not applicable for ionothermal synthesis [31]. The CTF was prepared in analogy to the literature [32]. Biphenyl (2.313 g, 15 mmol), cyanuric chloride (1.844 g, 10 mmol), and anhydrous AlCl_3_ (6.000 g, 45 mmol) were refluxed in 500 mL of DCM for 16 h. After filtration, the product was washed 15 min for each step with water (3 × 50 mL) and methanol (3 × 50 mL). Further washing steps were carried out with THF (3 × 50 mL) and acetone (3 × 50 mL). The product was dried at 120 °C for 24 h and was milled with a shaker mill (Retsch, MM301) with a frequency of 30 Hz for 15 min to obtain a fine powder (yield: 2.87 g; 94%).

### 2.3. Membrane Preparation

All membranes were prepared by solution casting. The filler loadings of CTF-biphenyl refer to the combined mass of the polymer and filler according to Equation (1):

(1) $$Filler\;loading\;\left[\mathrm{wt}\%\right]\;=\;\frac{m_{filler}}{m_{polymer}\;+\;m_{filler}}\;\times \;100\%$$

Matrimid^®^ was dried for 5 days at 80 °C before usage. For the pure Matrimid^®^ membranes, 400 mg of the polymer were dissolved in 5 mL of DCM and stirred for 24 h before casting. The CTF/Matrimid^®^ MMMs were prepared as follows [33]: Matrimid^®^ (400 mg) was dissolved in 3.5 mL of DCM and stirred for 24 h. Meanwhile, CTF-biphenyl (35 mg for 8 wt%, 76 mg for 16 wt%, and 126.5 mg for 24 wt%) was dispersed in 4.5 mL of DCM and stirred for 24 h. The CTF-biphenyl dispersion was ultrasonicated (VCX 750 Sonics; Microtip 630-0419) with an amplitude of 20% three times for 15 min each. After each 15 min ultrasonication procedure, the dispersion was stirred for 30 min. Then, part of the polymer solution (0.33 mL for 8 wt%, 0.72 mL for 16 wt% and 1.20 mL for 24 wt%) was added to the CTF-biphenyl dispersion followed by another 24 h of stirring. After the same three 15 min ultra-sonification steps were repeated, the remaining polymer solution was added and stirred for 1 h. For all membranes, the solution casting and drying was carried out under the same conditions: The mixtures were cast into metal rings placed on a flat glass surface. In order to achieve a controlled evaporation of DCM, an inverted funnel, covered with a paper tissue, was placed above the metal ring. When the DCM was evaporated, the membrane was cut out with a scalpel and dried in a vacuum oven (150 °C, 20 mbar) overnight. For the preparation of the IL-containing membranes, see Section 3 in the Supplementary Materials. All membranes were measured after drying in the vacuum oven and no further treatment with alcohol was performed. In the literature, such an alcohol treatment is sometimes performed to increase the permeability for a certain time period [34].

### 2.4. Instrumentation and Characterization Methods

Attenuated total reflection infrared spectroscopy (ATR-IR, Platinum ATR-QL, diamond) in the range from 4000 cm^–1^ to 500 cm^–1^ was measured on a Tensor 37 (Bruker). A vario MICRO cube (elementar) was used to perform elemental analysis (C, H, N, S). Scanning electron microscopy (SEM) images were obtained by a JSM-6510LV (Jeol) with a LaB_6_ cathode (20 keV) after coating of the sample with gold by a JFC 1200 (Jeol) coater. All membranes were broken through freeze-fracturing after cooling in liquid nitrogen to obtain cross-section images. A TG 209 F3 Tarsus (Netzsch) was used for thermogravimetric analysis (TGA). Measurements were carried out under synthetic air in a range from 25 °C to at least 700 °C with a heating rate of 5 K/min. The water content of the IL was quantified with coulometric Karl-Fischer titration (KFT) using an AQUA 40.00 (Analytik Jena/ECH) with a headspace module at 170 °C. ^1^H and ^13^C-NMR spectra were recorded with an Avance III spectrometer (Bruker) operating at 300 and 75 MHz, respectively. Density determination was carried out with an AccuPyc 1330 helium pycnometer (Micromeritics). A determination was performed in triplicate with 5 measured values each. For the density determination (Equation (2)), the total pore volume from the N_2_ sorption was considered due to the fact that helium does not fill the pores of the CTF at room temperature:

(2) $$\rho_{CTF}\;=\;\frac{V_{He}\;+\;V_{pores}}{m_{CTF}}$$

Nitrogen sorption measurements were performed on an Autosorb-6 (Quantachrome). Brunauer–Emmett–Teller (BET) surface areas were calculated from the adsorption isotherms by applying multipoint analysis in the range of 0.05 to 0.3 p/p_0_ with a correlation coefficient of minimum r = 0.999994. CO_2_ and CH_4_ sorption measurements were performed with a sorption analyzer Autosorb-iQ MP (Quantachrome) and the resulting isotherms were fitted with the Toth model (3Psim software version 1.1.0.7.) by applying Equation (3), where q refers to the loading in mmol/g, K stands for the affinity constant with the unit 1/bar and t refers to the heterogeneity exponent:

(3) $$q_{eq}\;=\;q_{max}\;\times \;\frac{K\;\times \;p}{{\left(1\;+\;{\left(K\;\times \;p\right)}^{t}\right)}^{\frac{1}{t}}}$$

All samples were activated before sorption measurements by degassing in vacuum at 120 °C for 8 h. CO_2_/CH_4_ mixed-gas separation was carried out on an OSMO inspector (provided by Convergence Industry B.V.) connected to an Agilent 490 Micro GC (Agilent Technologies) with a fused silica column PoraPLOT Q. A schematic drawing of the experimental setup has been presented in a previous publication [23]. To ensure that all membranes have an area of 11.3 cm^2^ while measuring, the membranes were placed in a permeation module and fixed with a Viton O-ring with an inner diameter of 3.6 cm. The mixed-gas separation experiments were carried out with a transmembrane pressure of 3 bar at 25 °C and were checked every 30 min with GC measurements until an equilibrium state was reached (after about 5–8 h). Once the membranes were equilibrated, the characteristic permeability was calculated from at least the last three recorded measurements. The feed gas consisted of CO_2_/CH_4_ in a volume ratio of 1:1 and helium was used as sweep gas. Each membrane was prepared and measured twice. The permeability *P* in Barrer ($1\;\mathrm{Barrer}\;=\;10^{-10}{\mathrm{cm}}^{3}\;\left(STP\right)\;\times \;\mathrm{cm}\;\times \;{\mathrm{cm}}^{-2}\;\times \;s^{-1}\;\times \;\mathrm{cm}{\mathrm{Hg}}^{-1}$) [35] was calculated according to the following Equation (4):

(4) $$P\;=\;\frac{x_{A}\;\times \;Q_{He}\;\times \;d}{x_{He}\;\times \;A\;\times \;\left(p_{2}\;\times \;x_{A}^{f}\;-\;p_{1}\;\times \;x_{A}\right)}$$

where $x_{A}$, $Q_{He}$, and d are the molar fraction of the gas A, the volumetric flow rate of the sweep gas helium, and the thickness of the membrane, measured at 10 different points with a micrometer screw, respectively. $x_{He}$, A, $p_{2}$, $x_{A}^{f}$, and $p_{1}$ are the molar fraction of the sweep gas (permeate), the area of the membrane, the feed pressure, the molar fraction of the gas A in the feed, and the permeate pressure, respectively. The mixed-gas selectivity (Equation (5)) of two gases (A and B) was calculated from their molar fractions (*x*) on the permeate side divided by their molar fractions on the feed side:

(5) $$\alpha_{A,B}\;=\;\frac{{\left(x_{A}/x_{B}\right)}_{permeate\;side}}{{\left(x_{A}/x_{B}\right)}_{feed\;side}}$$

## 3. Results and Discussion

### 3.1. Characterization of CTF-Biphenyl

The successful formation of CTF-biphenyl was confirmed by ATR-IR (Figure S1). The bands at 1704 cm^−1^ (w) and 1606 cm^−1^ (w) can be assigned to C=C bond stretching vibrations and the bands at 1511 cm^−1^ (s), 1377 cm^−1^ (w), and 1254 cm^−1^ (m) are due to C–N bond stretching vibrations of the triazine unit [36]. Elemental analysis (Table S1) showed a lower nitrogen content than calculated for the idealized structure, which is a common phenomenon concerning CTFs synthesized by Friedel–Crafts alkylation or by other methods like trifluoromethanesulfonic acid-catalyzed reactions or ionothermal synthesis [32,36,37]. Due to the hygroscopic nature of CTFs with a water uptake of up to ~20 wt% at 50–60% air humidity [32,38], the nitrogen and carbon wt% is already lowered due to adsorbed water during probe handling. For a comparison with other CTF structural analogs to CTF biphenyl, see Section 1 in the Supplementary Materials. TGA measurements (Figure S2) confirmed a thermal stability up to 320 °C under synthetic air and SEM images of CTF-biphenyl (Figure S3) showed particles with a mainly spherical shape. N_2_-sorption (Figure S4a) of CTF-biphenyl revealed a transition from Type I to Type II isotherm for the adsorption from low to high relative pressure. The pronounced uptake at low p/p_0_ is associated with the filling of micropores. Desorption featured an H4 loop, which is often found with aggregates of micro-mesoporous carbons [39]. The isotherm and hysteresis type are in accordance with the observations from Lim et al., who synthesized the same CTF-biphenyl via Friedel–Crafts alkylation [40]. The CTF synthesized in this study exhibited a higher BET surface area of 940 m^2^/g with a total pore volume of 0.53 cm^3^/g (literature 646 m^2^/g and 0.31 cm^3^/g [40]). The pore size distribution was obtained by applying quenched solid density functional theory (QSDFT), which is favorable for disordered micro/mesoporous carbon materials [41]. The slit pore, equilibrium model (N_2_ at 77 K on carbon) was utilized and revealed pore diameters mainly at 9 and 13 Å, with smaller volume contributions up to 40–50 Å (Figure S4b). Further, CO_2_ and CH_4_ sorption of CTF-biphenyl (Figure S5) at 298 K resulted in CO_2_ and CH_4_ uptakes of 1.87 mmol/g (at 0.96 bar) and 0.55 mmol/g (at 0.97 bar). The CO_2_ and CH_4_ sorption isotherm fit with the Toth model (Table S3) allowed ideal adsorbed solution theory (IAST) calculations to be carried out, yielding a CO_2_/CH_4_ selectivity of 10.5 at 1 bar pressure for a 50:50 (v:v) gas mixture (Figure S6).

### 3.2. Characterization of CTF-Biphenyl/Matrimid^®^ MMMs and Their Gas Separation Studies

**Figure 1:**
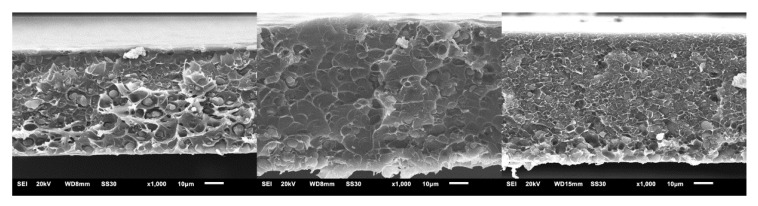
Cross-section SEM images of 8 wt% (**left**), 16 wt% (**middle**), and 24 wt% (**right**) CTF/Matrimid^®^ MMMs. The cross-sections were formed by breaking the membrane through freeze-fracturing after cooling in liquid nitrogen.

All CTF-based MMMs were analyzed by cross-section SEM images to confirm the incorporation of the filler materials as well as to insure the absence of defects in the MMMs. Figure 1 shows the cross-section SEM images of the CTF/Matrimid^®^ MMMs. The absence of heavy atoms in the CTF and no distinct particle shape yielded a low contrast between the filler and polymer. Unfortunately, the SEM images did not prove very expressive concerning filler–polymer interface compatibility or aggregation. Only for the MMM with 8 wt% can relatively more interfacial voids be observed as compared to the MMMs with higher filler contents. All of the CTF-based MMMs have no major defects that would cause a loss of selectivity in the permeation experiments.

**Figure 2:**
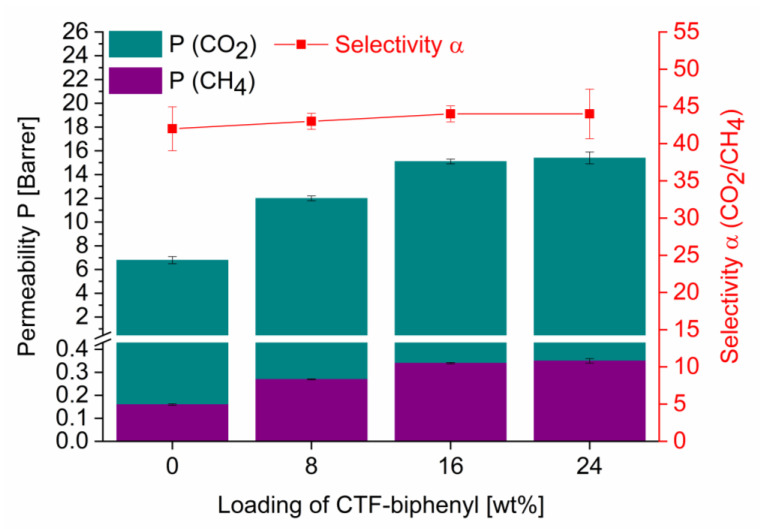
CO_2_ and CH_4_ permeabilities (*P*) and CO_2_/CH_4_ selectivities (α) for the pure Matrimid^®^ membranes and CTF/Matrimid^®^ MMMs.

The neat polymer membrane and the MMMs were further applied for CO_2_/CH_4_ mixed-gas separation experiments. For the CTF/Matrimid^®^ MMMs, CO_2_ permeability increased from 6.8 Barrer for the neat Matrimid^®^ membrane to 12.0 Barrer for 8 wt% of CTF-biphenyl. For a filler content of 16 wt% CTF-biphenyl, the CO_2_ permeability was raised further to 15.1 Barrer. With elevation of the filler content to 24 wt%, the permeability stayed essentially constant within the experimental error, reaching a value of 15.4 Barrer (Table 1 and Figure 2). The CH_4_ permeability of all membranes was below 0.35 Barrer. A constant permeability for 16 and 24 wt% was also observed for CH_4_. Consequently, the CO_2_/CH_4_ mixed-gas selectivities remained constant within the experimental error for all amounts of CTF-biphenyl applied. The higher the filler content, the lower the proportional increase in CO_2_ permeability. In conclusion, the 16 wt% MMM showed the optimal membrane performance considering its high absolute permeability value, constant selectivity, and the medium consumption of the filler material. 

The ratio of the permeability of the MMMs (*P_eff_*) and the permeability of the pure continuous phase (*P_c_*) can be predicted for different filler volume fractions (*ϕ_d_*). Different models, such as the Maxwell [42], Bruggeman [43], and Böttcher–Landauer model [44], for porous fillers can be applied. The simplified form of the Maxwell model for the case *P_d_ >> P_c_* (*P_d_*: permeability of the dispersed phase) is shown in the following Equation (6):

(6) $$\frac{P_{eff}}{P_{c}}\;=\;\frac{1\;+\;2\phi_{d}}{1\;-\;\phi_{d}}$$

At this point, it should be noted that the validity of the model is given for *ϕ_d_* up to a maximum of 0.2. For the case of *ϕ_d_* > 0.2, the Bruggeman model (Equation (7)) or the Böttcher–Landauer model (Equation (8)) can be applied:

(7) $$\frac{P_{eff}}{P_{c}}\;=\;\frac{1}{{\left(1-\phi_{d}\right)}^{3}}$$

(8) $$\frac{P_{eff}}{P_{c}}\;=\;\frac{1}{\left(1-3\phi_{d}\right)}$$

**Figure 3:**
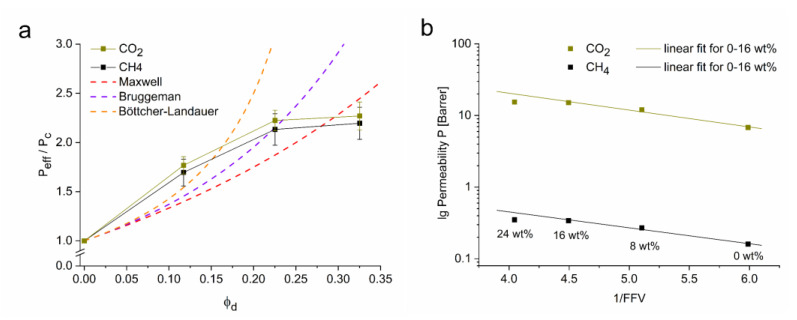
*P*_eff_/*P_c_* versus *ϕ_d_* for 0 wt%, 8 wt%, 16 wt%, and 24 wt% CTF-biphenyl/Matrimid^®^ MMMs in comparison to the Maxwell, Bruggeman, and Böttcher–Landauer model for porous fillers (**a**) and logarithm of permeability *P* versus 1/FFV for 0 wt%, 8 wt%, 16 wt%, and 24 wt% for CTF-biphenyl/Matrimid^®^ (right) MMMs (**b**).

The experimental results for *P_eff_*/*P_c_* versus *ϕ_d_* are given in Figure 3a. The filler contents of 8, 16, and 24 wt% CTF-biphenyl were converted into the volume fractions *ϕ_d_* of 0.12, 0.23, and 0.33, respectively. For this purpose, the determined density of CTF-biphenyl (0.79 g/cm^3^) and the density of Matrimid^®^ (1.20 g/cm^3^) [45] were used. The errors were calculated from the largest difference in permeability values that can occur in the relative relation of *P_eff_/P_c_*. Due to the constant CO_2_/CH_4_ selectivity, *P_eff_*/*P_c_* values are higher for the more permeable gas CO_2_. Comparing the experimental values with the models, it is noticeable that the permeabilities increase relatively more at lower *ϕ_d_* values. At 8 wt% MMM, the values for *P_eff_*/*P_c_* are higher than those predicted by all models. The 16 wt% MMM agrees well with the Bruggeman model. Due to the stagnation of the permeability values with 24 wt% filler, the permeability is much too low compared to the higher filler amount. 

Our explanation is an unexpected poor interfacial compatibility between the CTF and Matrimid^®^ giving a large interface volume and aggregation at higher filler content. At the relatively low filler content of 8 wt%, the filler particles still remain separated, but each particle would be surrounded by its interface volume. When the CTF particles aggregate at 24 wt%, the interface volume is relatively lower as the aggregated particles have a smaller surface-to-volume ratio than the separated particles at 8 wt%. The larger than normal interface volume at 8 wt% will then lead to an increase in permeability, which surmounts the expected increase from the fractional free volume (FFV) of the filler. Hence, the 8 wt% MMM surpasses the predicted *P_eff_*/*P_c_* ratio by the Maxwell model. The too low permeability of the 24 wt% MMM must be related to the aggregation effects such that less FFV of the filler is available and at the same time the aggregation also decreases the interface volume.

The influence of the free volume on the permeability can be illustrated by calculating the FFV. This dimensionless quantity can be obtained by multiplying the density and the pore volume of a material. The total FFV of a membrane (Equation (9)) is composed of the values of the polymer and the filler:

(9) $$FFV\;=\;FFV_{polymer}*\phi_{c}\;+\;FFV_{filler}*\phi_{d}$$

For the calculation, the literature value of 0.167 [46] was used for Matrimid^®^, and all other values were determined from the densities of the materials described above. Plotting the logarithm of permeability *P* against 1/FFV (Figure 3b), it becomes clear that a linear correlation between the permeability and the free filler volume only holds up to the 16 wt% MMM. Considering the logarithmic nature of the correlation, the permeability of the 24 wt% MMM is much too low for its assumed FFV. Instead, the FFV of the 24 wt% MMM corresponds to about the FFV of the 16 wt% MMM. For dense membranes, the permeability (*P*) can be described as the product of the diffusion coefficient (*D*) and solubility (*S*) [47]. In the case of glassy polymers, diffusivity contributes a significantly higher proportion to the overall permeability than solubility [48]. The free volume can therefore have a positive effect on diffusivity and thus on permeability to a certain extent. This relationship becomes evident when comparing the free volume in this membrane system with the permeability. In cases where less free volume was generated, the permeability is also relatively lower compared to the MMMs with a higher contribution of free volume. 

Although research on organic fillers in MMMs is increasing, there are few comparable materials embedded in a Matrimid^®^ matrix that are applied for CO_2_/CH_4_ separation. One of these materials is the azine-linked covalent organic framework ACOF-1. An equimolar CO_2_/CH_4_ mixture was used to determine the membrane performance of 8 and 16 wt% ACOF-1/Matrimid^®^ MMMs. With a feed gas pressure of 4 bar at 308 K, the CO_2_ permeability of the 8 and 16 wt% MMMs were found to be 9.6 and 15.3 Barrer in comparison to the pure matrix with a value of 6.8 Barrer. The selectivity was found to be constant [19]. The comparison also shows that a relatively high increase in CO_2_ permeability can be achieved with 8 wt% CTF-biphenyl while maintaining constant CO_2_/CH_4_ selectivity. For 16 wt% filler, the comparison with the literature shows an almost identical value. In comparison to other CTFs, such as CTF-fluorene and CTF-1 in Matrimid^®^ or in polysulfone (PSF), the membrane performance of CTF-biphenyl is slightly better or comparable both in permeability and selectivity [21,23]. Exceptions are the fillers SNW-1 and FCTF-1 in the polymer of intrinsic microporosity PIM-1, where already the PIM-1 gives rise to a CO_2_ permeability of several thousand Barrer, albeit at low CO_2_/CH_4_ selectivity (from single-gas measurements). For PIM-1, the filler still increases this permeability but at a similar ratio as seen for other MMMs [20,49]. An overview of organic filler materials in different polymer matrices for CO_2_/CH_4_ separation is given in Table S9.

In order to gain further insight into this system, investigations on the effect of a ternary component were conducted. Throughout the literature, the incorporation of an IL as a third component is described with highly variable effects on membrane performance (Table S6). Due to the high solubility of CO_2_ in ILs [24,25,26] and noted positive effects of ILs on membrane performance [27,28,29], we also investigated the incorporation of the IL 1-butyl-3-methylimidazolium bis(trifluoromethanesulfonyl)imide, [BMIm][NTf_2_] in the CTF/Matrimid^®^ MMMs (see Section 3 and Section 4 in the Supplementary Materials). The introduction of the IL as a ternary component showed no improvement in permeability over the CTF/Matrimid^®^ system. In a few membranes, the selectivity was raised with the IL to about 50–52 compared to about 44 for CTF/Matrimid^®^ (Table S8 vs. Table 1).

## 4. Conclusions

CTF-biphenyl was synthesized via Friedel–Crafts-alkylation from cyanuric chloride and biphenyl to be applied as porous filler material in Matrimid^®^ matrices. CO_2_/CH_4_ mixed-gas measurements of the prepared CTF/Matrimid^®^ MMMs resulted in an increase in CO_2_ permeability, while maintaining constant selectivity. For the MMMs with 8 wt% CTF, the CO_2_ permeabilities could be elevated from 6.8 Barrer for the pure polymer to 12.0 Barrer. Comparison with permeability models showed that these MMMs exhibited a much higher rise in permeability than expected, accompanied by a relatively higher free volume compared to the MMMs with higher filler contents. With 16 wt% CTF-biphenyl, the CO_2_ permeability was increased to 15.1 Barrer, which is in good accordance with the Bruggeman model. A further increase of the filler content to 24 wt% resulted in a stagnation in CO_2_ permeability, indicating that the optimum filler content should be used up to a maximum of 16 wt%. Even though CTFs are purely organic fillers, the comparison of the permeability of the CTF/Matrimid^®^ MMMs to theoretical models pointed at unexpected problems of the interface compatibility between the CTF filler and Matrimid^®^ matrix. The CTF filler enhanced the CO_2_/CH_4_ separation performance but in a way that necessitated a closer look. The success of CTF fillers depends strongly on the interface compatibility. In follow-up studies, we will address this interface problem.

## Figures and Tables

**Table 1:** Gas permeabilities (*P*) and mixed-gas selectivity factors (α) of the pure Matrimid^®^ membrane and CTF/Matrimid^®^ MMMs ^1^.

CTF-Biphenyl [wt%]	P CO_2_ [Barrer]	P CH_4_ [Barrer]	α CO_2_/CH_4_
0 (neat Matrimid^®^)	6.8 ± 0.3	0.16 ± 0.01	42 ± 1
8	12.0 ± 0.2	0.27 ± 0.01	43 ± 1
16	15.1 ± 0.2	0.34 ± 0.01	44 ± 1
24	15.4 ± 0.5	0.35 ± 0.01	44 ± 1

^1^ The errors for the permeability (*P*) and for the selectivity (α) were taken from the range of two measurements.

## Data Availability

The data presented in this study are available on request from the corresponding author.

## Supplementary Materials

The following are available online at https://www.mdpi.com/article/10.3390/membranes11100795/s1.
