# Supplementary material for: Biphenyl-Based Covalent Triazine Framework/Matrimid® Mixed-Matrix Membranes for CO2/CH4 Separation
Source: Membranes (Basel). 2021 Oct 19;11(10):795. doi: 10.3390/membranes11100795 (PMC8539902; doi:10.3390/membranes11100795)
Supplement: Supplementary file 1 [file membranes-11-00795-s001.zip › membranes-1396500-supplementary.pdf]

Supplementary Materials

Biphenyl-based covalent triazine framework/Matrimid® mixed-matrix membranes for CO_2/CH_4 separation

Stefanie Bügel,[1] Quang-Dien Hoang,[1] Alex Spieß,[1] Yangyang Sun,[1] Shanghua Xing,[1,2] Christoph Janiak*[1,2]

[1] Institut für Anorganische Chemie und Strukturchemie, Heinrich-Heine-Universität Düsseldorf, D-40204 Düsseldorf, Germany

[2] Hoffmann Institute of Advanced Materials, Shenzhen Polytechnic, 7098 Liuxian Blvd, Nanshan District, Shenzhen 518055, China

*Corresponding author: Christoph Janiak
Email: stefanie.buegel@uni-duesseldorf.de; quang-dien.hoang@uni-duesseldorf.de; alex.spiess@hhu.de; yasun100@hhu.de; shanghua.xing@hhu.de; janiak@hhu.de

Content

1. Characterization of CTF-biphenyl 3

1.1. Infrared spectroscopy (IR) 3

1.2. Elemental analysis 3

1.3. Thermogravimetric analysis (TGA) 4

1.4. Scanning electron microscopy (SEM) 5

1.5. N_2-sorption 5

1.6. CO_2 and CH_4 sorption 5

1.7. Ideal adsorbed solution theory (IAST) 6

2. Synthesis and characterization of [BMIm][NTf_2] 7

2.1. Synthesis 7

2.2. ^{1}H-NMR 8

2.3. ^{13}C-NMR 9

3. Preparation and characterization of [BMIm][NTf_2]@CTF-biphenyl and IL-containing membranes 9

3.1. Preparation of [BMIm][NTf_2]@CTF-biphenyl 9

3.2. Elemental analysis 10

3.3. TGA 10

3.4. N_2-sorption 11

3.5. Preparation of IL-containing membranes 11

4. Characterization of membranes 12

4.1. Membrane thickness 12

4.2. Effect of [BMIm][NTf_2] as ternary component. 12

4.3. Cross-section SEM images of ternary MMMs 15

4.4. Gas permeabilities (*P*) and mixed-gas selectivity factors (α) 16

4.5. Comparison of binary MMMs with literature 17

References 18

1. Characterization of CTF-biphenyl

1.1. Infrared spectroscopy (IR)

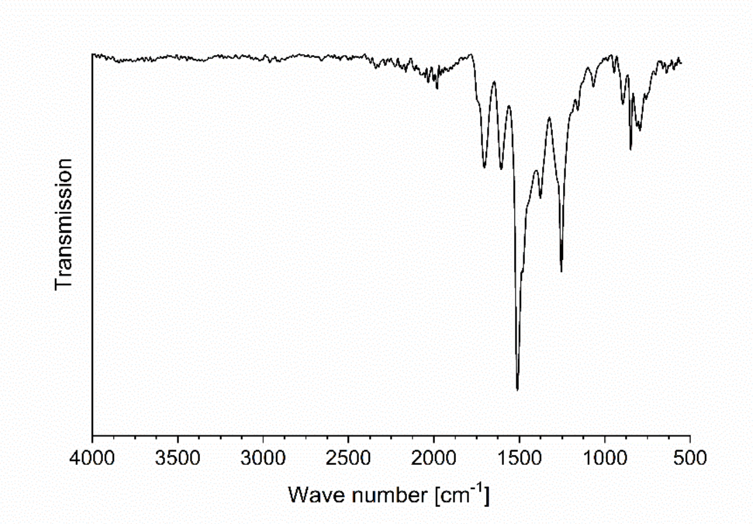

Figure S1. IR spectrum of CTF-biphenyl.

1.2. Elemental analysis

The final composition of CTFs strongly depends on the synthesis conditions. In the case of biphenyl-based CTFs, an optimal C/N ratio cannot be obtained using Friedel-Crafts synthesis, ionothermal synthesis or Suzuki coupling (Table S1). Only the synthesis under Brønsted acidic conditions shows good agreement with the theoretically calculated values. However, since the application of the materials for gas separation is the focus of this work, an appropriate porosity is of crucial importance. Since the BET-surface areas of the CTFs produced under Brønsted acidic conditions are generally lower [1,2], a different method must be employed. The ionothermal method generally produces CTFs with higher BET surface areas. The synthesis temperature plays a crucial role at this point. An increase in temperature leads to the generation of more defects, which increase the porosity but lead to a lower nitrogen content [3]. Another problem is the production of CTFs on a larger scale, which so far can best be solved with Friedel-Crafts alkylation. In a previous work, different synthesis conditions were investigated concerning Friedel-Crafts synthesis [4]. The use of cyanuric chloride in excess resulted in higher nitrogen content, but lower BET surface area. The optimal conditions for a CTF with the linker fluorene were transferred to the synthesis of CTF-biphenyl. A too low nitrogen content can be attributed to the formation of polymer chains through C-C bond formation of biphenyl units via the Scholl reaction [5,6], which would result in a relative reduced incorporation of triazine units. The remaining mass fraction can be explained by residues of aluminum species

from the catalyst $AlCl_3$, as well as by an undetectable oxygen content. The C/H ratio is somewhat lower than theory, that is the hydrogen wt% is higher than expected. This can be due to the presence of adsorbed water/moisture during sample handling and storage before CHN analysis, as discussed in [7,8,9]. Subsequently, the adsorbed water content will also add to the lower than expected nitrogen wt%.

Table S1. Elemental analysis of CTF-biphenyl and other biphenyl-based CTFs prepared by different synthesis methods.

Compound	Synthesis	C [wt%]	H [wt%]	N [wt%]	C/N ratio	C/H ratio	BET [m^2/g]	Ref.
Theoretical[a]		82.33	3.95	13.72	6.00	20.84	-	-
CTF-biphenyl	Friedel-Crafts	71.29	3.80	6.92	10.30	18.76	940	This work
CTF-DCBP	Ionothermal	84.20	2.18	5.41	15.56	38.62	2475	[10]
CTF-2	Suzuki	69.26	4.84	9.00	7.70	14.31	200-400	[11]
CTF-2	Brønsted acidic	75.88	3.81	12.21	6.21	19.92	560	

[a]For idealized structure, see Scheme 1.

1.3. Thermogravimetric analysis (TGA)

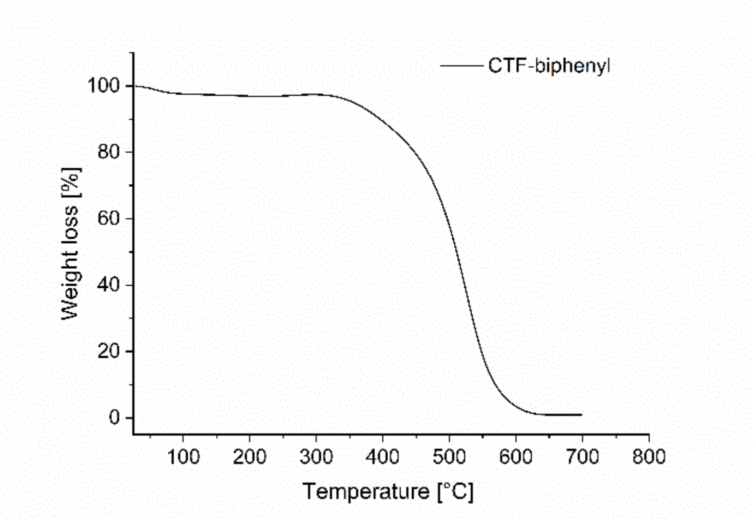

Figure S2: TGA curve of CTF-biphenyl measured under synthetic air

1.4. Scanning electron microscopy (SEM)

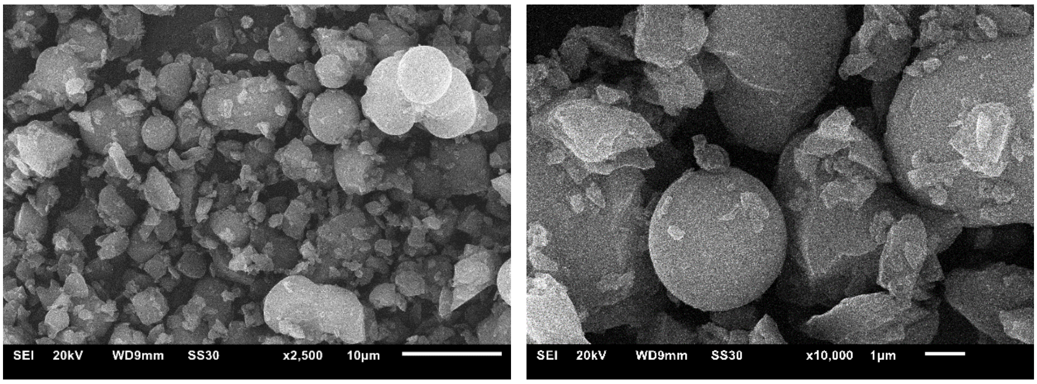

Figure S3. SEM images of CTF-biphenyl.

1.5. N_2-sorption

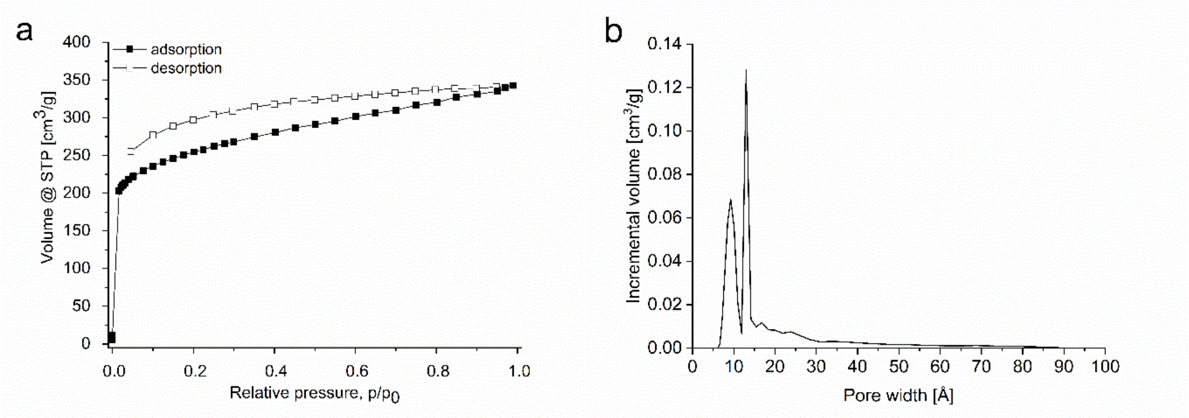

Figure S4. N_2-sorption isotherm (a) and pore size distribution (b, by QSDFT) of CTF-biphenyl.

1.6. CO_2 and CH_4 sorption

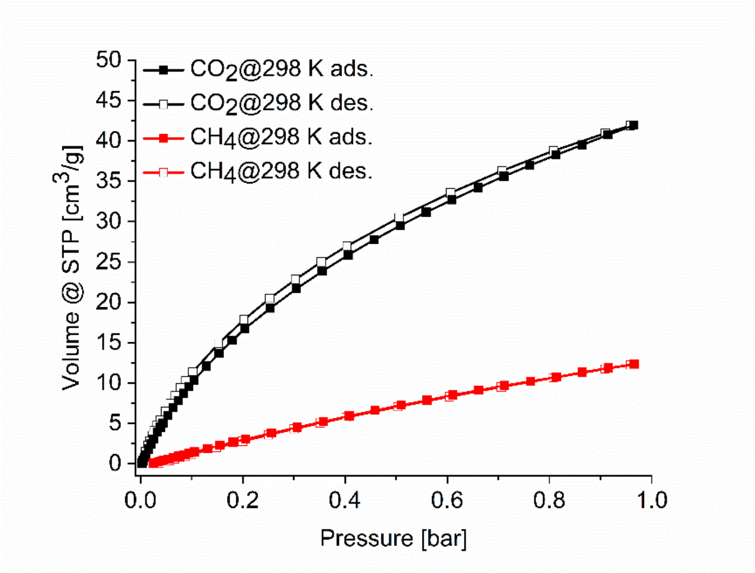

Figure S5. CO_2 and CH_4 sorption isotherms of CTF-biphenyl at 298 K.

Table S2. Comparison of CO_2 uptake capacities and BET-surface areas of different biphenyl-based CTFs (1 bar, 298 K).

CTF material	Synthesis	CO_2 uptake (1 bar, 298 K)	BET surface area, N_2 at 77K [m^2/g]	Reference
CTF-biphenyl	Friedel-Crafts	1.87 mmol/g[a]	940	This work
CTF-2	Brønsted acidic	1.25 mmol/g	560	[11]
CTF-2 Suzuki	Suzuki	0.19 mmol/g	209	
Material 3	Friedel-Crafts	41 cm^3/g	646	[12]

[a] at 0.96 bar

1.7. Ideal adsorbed solution theory (IAST)

The CO_2 and CH_4 isotherms (298 K) of CTF-biphenyl were fitted with the Toth model.

Table S3. Parameters for Toth fitting.

Gas	Temperature [K]	Model	R^2	Affinity constant K [1/bar]	Maximal loading q_{max} [mmol/g]	Heterogeneity exponent t
CO_2	298	Toth	0.9999	1.126	6.246	0.553
CH_4	298	Toth	0.9990	0.771	0.841	2.749

The selectivity of CTF-biphenyl for a binary (50:50; v:v) mixture of the gases CO_2/CH_4 was calculated by applying the ideal adsorbed solution theory, IAST. Figure S6 depicts the CO_2/CH_4 selectivity as a function of the pressure.

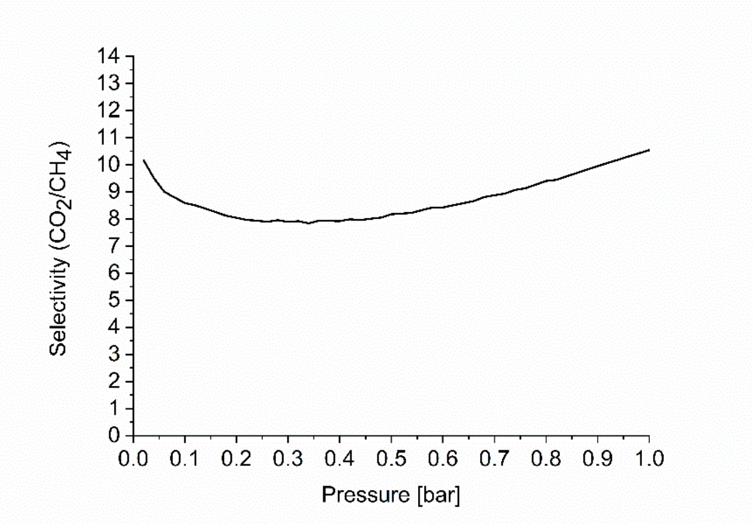

Figure S6. IAST selectivity of CTF-biphenyl for a binary (50:50; v:v) mixture of the gases CO_2/CH_4 at 298 K.

2. Synthesis and characterization of [BMIm][NTf_2]

2.1. Synthesis

[BMIm]Cl was synthesized with 1-methylimidazole (24.63 g, 0.3 mol) and 1-chlorobutane in equimolar proportions (27.77 g, 0.3 mol) via a microwave reaction [13]. The educts were stirred and treated for 1 h at 150 °C and 500 W in a microwave reactor (CEM, Mars 6). The resulting [BMIm]Cl was added dropwise to ice-cold ethyl acetate. After decanting, the solid [BMIm]Cl was dried under vacuum (yield: 47.8 g; 91 %). 1-Butyl-3-methylimidazolium bis(trifluoromethanesulfonyl)imide, [BMIm][NTf_2] was obtained by anion exchange. For the metathesis reaction, [BMIm]Cl (43.67 g, 0.25 mol) and lithium bis(trifluoromethane-sulfonyl)imide (71.77 g, 0.25 mol) were stirred in water for 24 h. The aqueous phase was extracted three times with DCM (200°mL) and subsequently washed with water until the washing solution had a neutral pH and was chloride free (tested with 0.1 mol/L silver nitrate solution). After adding a tip of a spatula of activated carbon, the suspension was stirred overnight and then filtered through acidic aluminum oxide (67 g in a column of 3.2 cm diameter). The product was dried under vacuum (yield: 91.7 g; 88 %). The formation of [BMIm][NTf_2] was confirmed by ^{1}H-NMR and ^{13}C-NMR (Figure S7 and Figure S8). The water content determined by Karl-Fischer titration (KFT) was less than 10 ppm.

2.2. ^{1}H-NMR

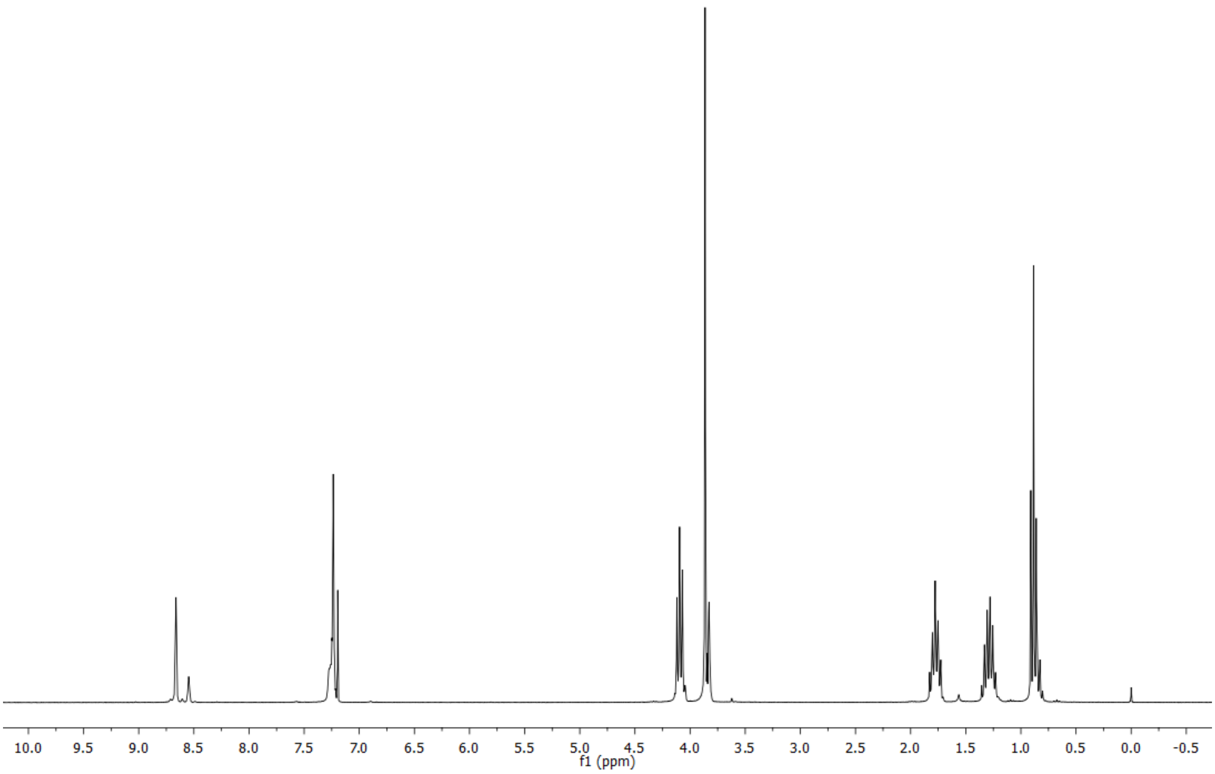

Figure S7. ^{1}H-NMR spectrum of [BMIm][NTf_2] in $CDCl_3$.

^{1}H-NMR (300 MHz, $CDCl_3$): δ = 8.66 (s, -CH_3, 1H), 7.24 (p, J = 2.0 Hz, 2 x -CH, 2H), 4.10 (t, J = 7.5 Hz, -CH_2, 2H), 3.86 (s, -CH_3, 3H), 1.78 (p, J = 7.5 Hz, -CH_2, 2H), 1.29 (h, J = 7.4 Hz, -CH_2, 2H), 0.89 (t, J = 7.4 Hz, -CH_3, 3H).

2.3. ^{13}C-NMR

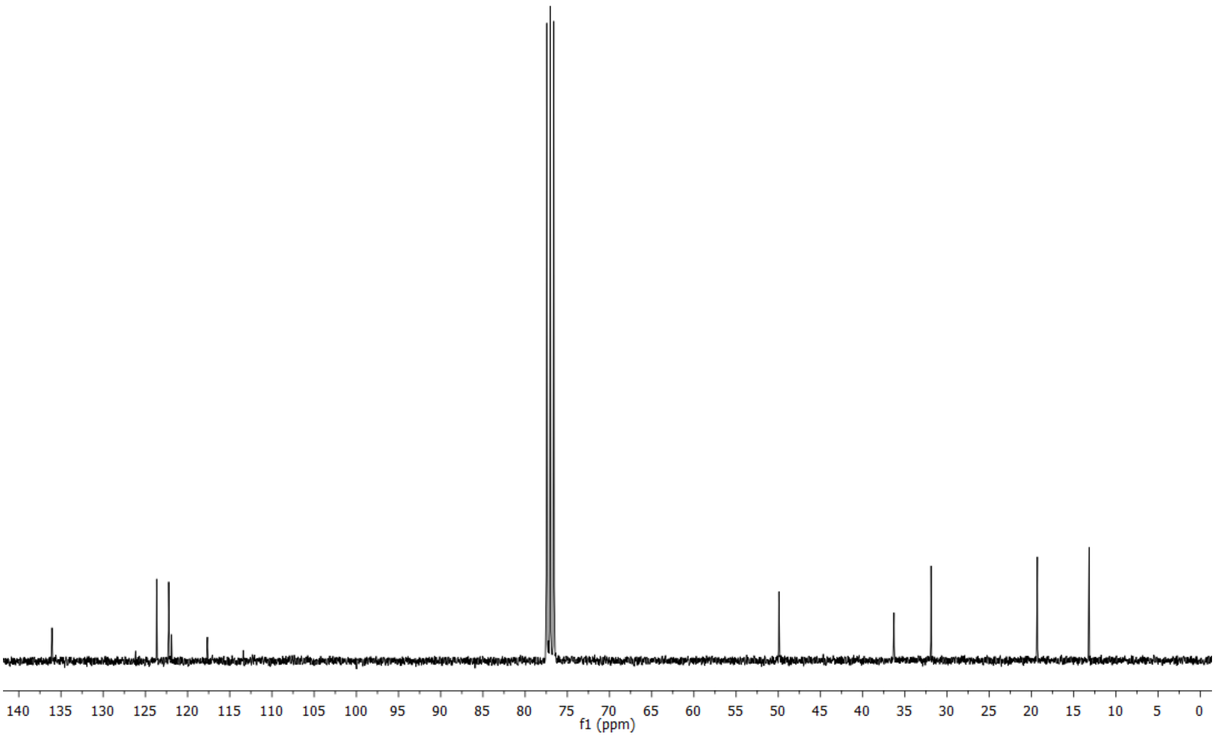

Figure S8. ^{13}C-NMR spectrum of [BMIm][NTf_2] in $CDCl_3$.

^{13}C NMR (75 MHz, $CDCl_3$): δ = 136.06 (s, N-CH-N), 123.66 (s, CH=C), 122.24 (s, CH=C), 117.65 (s, CF_3), 49.93 (s, N-CH_2-C), 36.30 (s, N-CH_3), 31.90 (s, CCH_2-C), 19.31 (s, CCH_2-C), 13.18 (s, C-CH_3).

3. Preparation and characterization of [BMIm][NTf_2]@CTF-biphenyl and IL-containing membranes

3.1. Preparation of [BMIm][NTf_2]@CTF-biphenyl

The IL 1-butyl-3-methylimidazolium bis(trifluoromethanesulfonyl)imide, [BMIm][NTf_2] was incorporated in the pores of CTF-biphenyl by post-impregnation in analogy to the preparation of [BMIm][NTf_2] in the pores of COF-300 [14]. [BMIm][NTf_2] (1.50 g) were dissolved in 95 mL of methanol and stirred for 4 h. CTF-biphenyl (500 mg) was slowly added while stirring. After sonification for 1 h, the dispersion was centrifuged (4 x 5 min, 8000 rpm) and washed with methanol (15 mL). The product was dried at 120 °C for 24 h (yield: 510 mg).

3.2. Elemental analysis

The presence of the IL in the pores of the CTF was confirmed by elemental analysis (Table S4). From the sulfur content of 0.86 wt% of the elemental analysis the IL content of IL@CTF was calculated as 5.62 wt% ([BMIm][NTf_2], $C_{10}H_{15}F_6N_3O_4S_2$, M = 419.36 g/mol) by applying equation 1:

$$IL\ wt\% = \frac{S\ wt\% \times M(IL)}{2 \times M(S)} \qquad (1)$$

Table S4. Elemental analysis of [BMIm][NTf_2]@CTF-biphenyl.

Compound	C [wt%]	H [wt%]	N [wt%]	S [wt%]
[BMIm][NTf_2]@CTF-biphenyl	67.71	3.39	6.34	0.86

3.3. TGA

TGA measurements were carried out under synthetic air to ensure that both the CTF and the IL@CTF composite had a thermal stability of at least 150°C and could therefore be applied as a filler material for membrane preparation. The measurement showed a thermal stability of IL@CTF up to 220 °C and confirmed the expected lower thermal stability of the composite in comparison to the pure CTF [15].

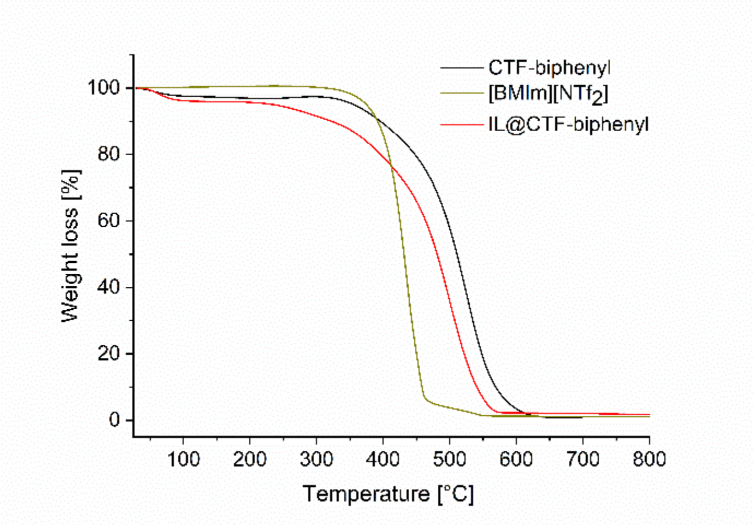

Figure S9. TGA curve of [BMIm][NTf_2]@CTF-biphenyl in comparison to curves of CTF and IL. Measured under synthetic air.

3.4. N_2-sorption

N_2-sorption isotherms (Figure S10a) of IL@CTF supported the incorporation of [BMIm][NTf$_2$] into the pores of CTF-biphenyl through the reduction of the BET surface area from 940 m²/g to 502 m²/g accompanied with a lowering of the total pore volume from 0.53 cm^3/g to 0.26 cm^3/g. A comparison of the pore size distributions in Figure S10b indicated that only the smaller CTF micropores with a pore diameter around 9 Å remained unfilled while the pores above a diameter of 11 Å had essentially vanished. The filling of only the larger pores is in agreement with the dimensions (referred to van der Waals radii) of the IL cation with a length of about 11.4 x 5.5 x 2.8 Å and the IL anion with a length of about 10.9 x 5.1 x 4.7 Å [16].

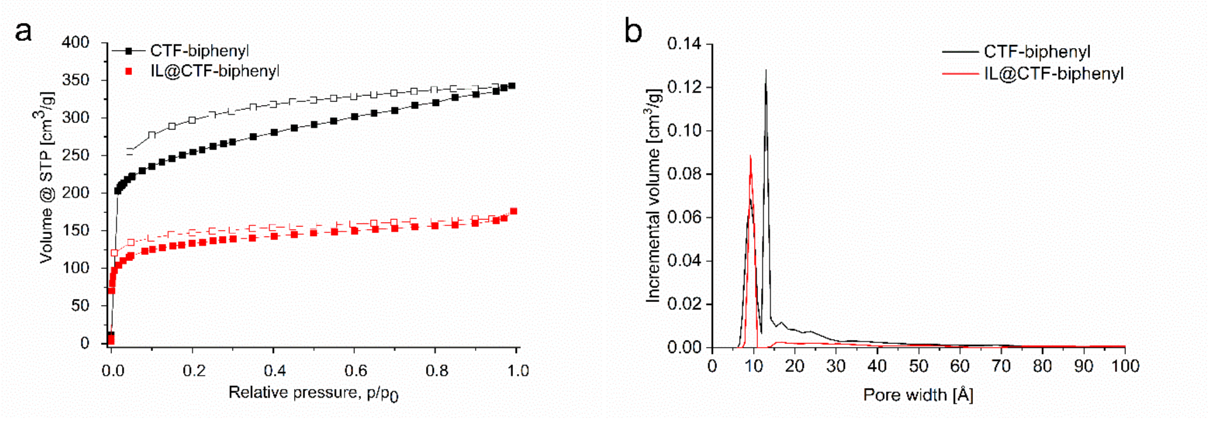

Figure S10. N_2-sorption isotherm (a) and pore size distribution (b, by QSDFT) of [BMIm][NTf$_2$]@CTF-biphenyl in comparison to the pure CTF.

3.5. Preparation of IL-containing membranes

The IL/Matrimid® membranes were prepared by direct physical mixing in DCM in analogy to the pure Matrimid® membranes. The IL@CTF/Matrimid® MMMs were prepared according to the same procedure by using IL@CTF (35 mg for 8 wt%, 76 mg for 16 wt% and 126.5 mg for 24 wt%) instead of CTF-biphenyl. As noted in the main text, the filler loadings of CTF-biphenyl and IL refer to the combined mass of the polymer and filler according to equation 1. For CTF/IL/Matrimid® MMMs 5 wt% IL (21 mg, based on 400 mg polymer) was added to the Matrimid® DCM solution before the first 24 h of stirring. The further procedure with the addition of the CTF (35 mg for 8 wt%, 76 mg for 16 wt% and 126.5 mg for 24 wt%) was as described above. For all membranes the solution casting and drying was carried out under the same conditions: The mixtures were cast into metal rings placed on a flat glass surface. In order to achieve a controlled evaporation of DCM, an inverted funnel, covered with a paper tissue,

was placed above the metal ring. When the DCM was evaporated, the membrane was cut out with a scalpel and dried in a vacuum oven (150 °C, 20 mbar) overnight.

4. Characterization of membranes

4.1. Membrane thickness

Table S5. Average thickness of CTF-biphenyl/Matrimid®, [BMIm][NTf$_2$]/CTF-biphenyl/Matrimid® and [BMIm][NTf$_2$]@CTF-biphenyl/Matrimid® MMMs.

CTF-biphenyl content [wt%]	CTF-biphenyl/ Matrimid®	[BMIm][NTf$_2$]/ CTF-biphenyl/ Matrimid®	[BMIm][NTf$_2$] @CTF-biphenyl/ Matrimid®
	Average thickness [µm]		
8	49.5	44	44.5
16	72.5	55	67.5
24	75.5	68.5	72

4.2. Effect of [BMIm][NTf$_2$] as ternary component.

In addition to the binary membrane system CTF-biphenyl/Matrimid® another binary IL/Matrimid® and two ternary membrane systems were prepared. The resulting [BMIm][NTf$_2$]/Matrimid®, CTF-biphenyl/[BMIm][NTf$_2$]/Matrimid® and [BMIm][NTf$_2$]@CTF-biphenyl/Matrimid® MMMs will be denoted as IL/Matrimid®, CTF/IL/Matrimid® and IL@CTF/Matrimid® MMMs. In order to choose an adequate amount of the IL [BMIm][NTf$_2$] for the ternary MMMs with CTF-biphenyl and Matrimid®, first only IL and Matrimid® were used to produce binary MMMs. The incorporation of 5 wt% IL into the Matrimid® matrix decreased the permeability but led to an increase in selectivity from 42 for the pure Matrimid® membrane to 49 for the composite. As the IL loading was enhanced to 10 and 15 wt%, both selectivity and permeability decreased (Table S7). Based on this result, the ternary CTF/IL/Matrimid® MMMs were prepared with 5 wt% IL. In the MMM CTF/IL/Matrimid®, the IL was used as a filler material alongside the CTF with the expectation that the IL could serve as a surfactant [15]. In IL@CTF/Matrimid® the IL was encapsulated in the pores of the CTF to enhance the molecular sieving effect by reducing the pore size (Figure S10b). With the incorporation of 5 wt% IL ([BMIm][NTf$_2$]) as the third component, the IL/CTF/Matrimid® MMMs with 8 and 16 wt% CTF-biphenyl showed essentially no increase in permeability compared to the pristine polymer with 6.8 Barrer (Figure S11a). For 24 wt% CTF-biphenyl the CO_2 permeability was elevated to 11.1 Barrer. However, for the

IL/CTF/Matrimid® system, the permeabilities were lower than those of the binary CTF/Matrimid® MMMs, but an increase in selectivity to 50 for the 24 wt% MMM was observed. Based on these results, it can be assumed that no additional free volume was generated, but that the IL may have had a positive influence due to its good CO_2 affinity resulting in an increased CO_2/CH_4 selectivity. When the IL was incorporated into the pores of CTF-biphenyl and then IL@CTF was added as the filler material in the IL@CTF/Matrimid® MMMs an improvement in CO_2 permeability compared to the pristine polymer was observed. The CO_2/CH_4 selectivity was raised to 52 and 46 for the 16 and 24 wt% MMMs, respectively (Figure S11b) albeit at somewhat lower permeabilities than the binary CTF/Matrimid® MMMs.

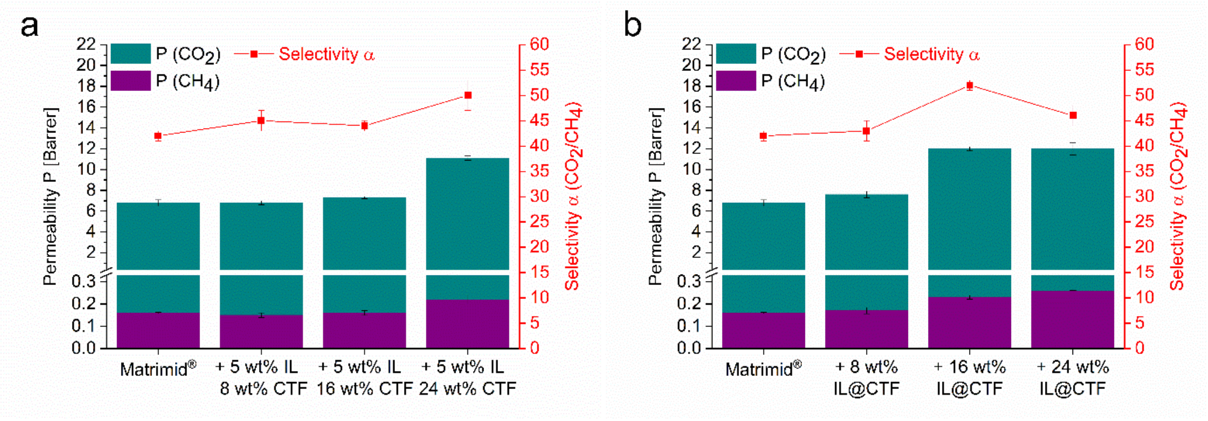

Figure S11. CO_2 and CH_4 permeabilities (*P*) and CO_2/CH_4 selectivities (α) for the pure Matrimid® membranes and IL/CTF-biphenyl/Matrimid® (a) and IL@CTF/Matrimid® (b) MMMs.

Literature reports suggest that incorporated ILs can have different effects on membrane performance. COF-300, synthesized by a condensation reaction of tetrakis(4-aminophenyl)methane and terephthalaldehyde, and the IL [BMIm][NTf_2] were incorporated into a Pebax® 1657 matrix by Zhao et al. [BMIm][NTf_2] in the pores of COF-300 could improve the CO_2 permeability from 81.7 Barrer to 242.1 Barrer with an elevation in CO_2/CH_4 selectivity from 18.8 to 39.5 [14]. Using the same polymer and IL, Li et al. prepared MMMs with the zinc-2-methylimidazolate framework ZIF-8 and investigated the performance with respect to CO_2/CH_4 separation. The highest CO_2 permeability value was achieved with 15 wt% of ZIF-8 in a binary MMM. The ternary system with additional [BMIm][NTf_2] in the pores of the filler material led to a decrease in CO_2 permeability, but an increase to 34.8 in CO_2/CH_4 selectivity [15]. The application of [BMIm][NTf_2] and ZSM-5 in a 6FDA-TeMPD matrix led to a decrease in CO_2 permeability from 1156 to 441 Barrer whilst the selectivity was only slightly increased from 18.6 to 21.1 [17].

Table S6. Comparison of CO_2 and CH_4 permeability and CO_2/CH_4 selectivity for ternary MMMs with the IL [BMIm][NTf$_2$].

Continuous phase	Filler	IL	P CO_2 [Barrer]	S CO_2/CH_4	Reference
Pebax® 1657[a]	-	-	81.7	18.8	[14]
Pebax® 1657[a]	COF-300 7 wt%	-	109.4	24.4	
Pebax® 1657[a]	COF-300 7 wt%	[BMIm][NTf$_2$] 3.8 wt%@COF	242.1	28	
Pebax® 1657[b]	-	-	72.5	18.1	[15]
Pebax® 1657[b]	ZIF-8[1] 15 wt%	-	(129)*	(19.5)*	
Pebax® 1657[b]	ZIF-8[1] 15 wt%	[BMIm][NTf$_2$] 16.5 wt%@ZIF	104.9	34.8	
6FDA-TeMPD[d,2]	-	-	1156 ± 96	18.6 ± 0.3	[18]
6FDA-TeMPD[d,2]	-	[BMIm][NTf$_2$] 10 ± 1 wt%	412 ± 67	23.9 ± 1.5	
6FDA-TeMPD[e,2]	ZSM-5[3] 15 wt%	[BMIm][NTf$_2$] 9 wt%	441 ± 17	21.1 ± 0.2	[17]

*information obtained from graph [a]mixed-gas CO_2/CH_4 (30/70 vol%); 1 bar feed pressure; 30 °C [b]single gas; 1 bar feed pressure; 25 °C [c]equimolar mixed-gas CO_2/CH_4; 6 bar feed pressure; 30°C [d]single gas; upstream pressure 75–77 cmHg; 35 °C [e]single gas; upstream pressure 10 atm; 35 °C; [1]zeolitic imidazolate framework; [2]polyimide derived from 4,4-hexa-fluoroisopropylidenediphtalicanhydride (6FDA)/2,3,5,6-tetramethyl-1,4-phenylenediamine(TeMPD); [3]zeolite socony mobil

In our CTF/Matrimid® membrane systems with IL as the ternary component, an improvement in both permeability and selectivity over the neat polymer could be achieved, but the binary CTF/Matrimid® MMMs still exhibited the best overall performance, when economic and environmental aspects are considered in addition to CO_2/CH_4 gas separation performance (IL is usually more expensive and not environmentally friendly due to the high fluorine content).The incorporation of ILs in MMMs is still largely empirical and further in-depth understanding on its polymer compatibility and its role is needed for a more knowledge-based approach.

In summary, two ternary CTF MMMs with the IL [BMIm][NTf_2] were prepared for the first time. The comparison to the binary CTF/Matrimid® MMMs, indicated that the incorporation of an IL as ternary component may not be beneficial for every MMM system.

We decided not to present the three MMM systems with IL in the manuscript because there is no clear improvement in membrane performance. The often-expected effect of ILs to increase membrane performance did not occur. We are critical of the incorporation in this case and can say that IL is not necessarily beneficial. Our first consideration was not to describe the IL MMMs, but we feel that the mention of not so successful experiments is an added value to shed light on certain topics from a different angle.

4.3. Cross-section SEM images of ternary MMMs

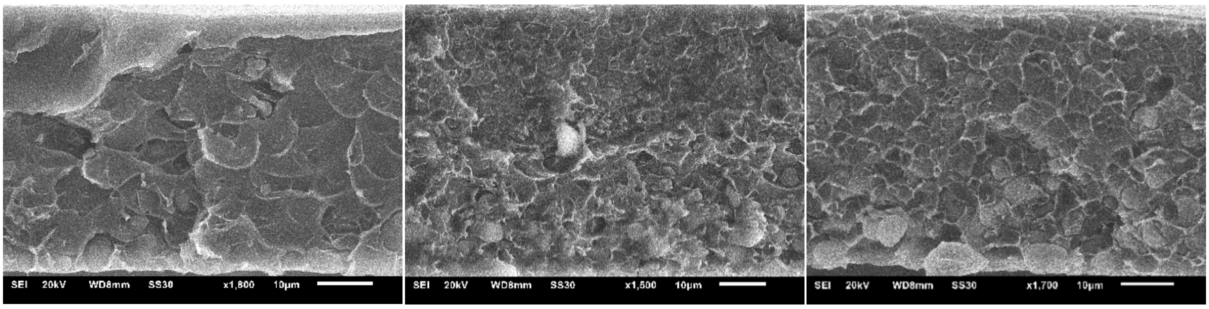

Figure S12. Cross-section SEM images of 8 wt%, 16 wt% and 24 wt% CTF in CTF/IL/Matrimid® MMM (with 5 wt% IL).

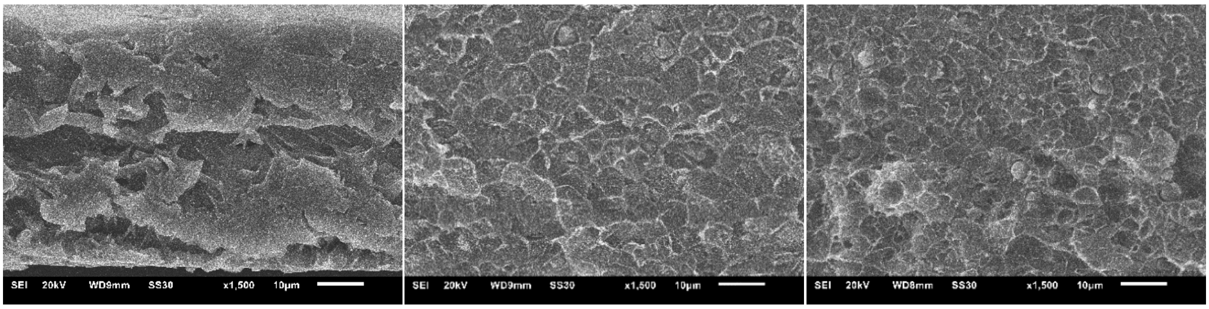

Figure S13. Cross-section SEM images of 8 wt%, 16 wt% and 24 wt% IL@CTF in IL@CTF/Matrimid® MMMs.

4.4. Gas permeabilities (*P*) and mixed-gas selectivity factors (α)

Table S7. Gas permeabilities (*P*) and mixed-gas selectivity factors (α) of $[BMIm][NTf_2]$/Matrimid® MMMs.[a]

IL $[BMIm][NTf_2]$ [wt%]	P CO_2 [Barrer]	P CH_4 [Barrer]	α CO_2/CH_4
5	5.5 ± 0.1	0.11 ± 0.01	49 ± 1
10	4.3 ± 0.2	0.11 ± 0.01	37 ± 1
15	3.7 ± 0.1	0.11 ± 0.01	34 ± 1

[a]The errors for the permeability (*P*) and for the selectivity (α) were taken from the range of two measurements.

Table S8. Gas permeabilities (*P*) and mixed-gas selectivity factors (α) of the ternary IL/CTF/Matrimid® and IL@CTF/Matrimid® MMMs.[a]

CTF-biphenyl [wt%]	IL [wt%]	combined filler [wt%]	P CO_2 [Barrer]	P CH_4 [Barrer]	α CO_2/CH_4
		IL/CTF/Matrimid®			
8	5	12	6.8 ± 0.2	0.15 ± 0.01	45 ± 2
16	5	20	7.3 ± 0.1	0.16 ± 0.01	44 ± 1
24	5	27	11.1 ± 0.2	0.22 ± 0.02	50 ± 3
		IL@CTF/Matrimid®			
7.6	0.48	8	7.6 ± 0.3	0.17 ± 0.01	43 ± 2
15.2	1.05	16	12.0 ± 0.2	0.23 ± 0.01	52 ± 1
23.0	1.74	24	12.0 ± 0.6	0.26 ± 0.01	46 ± 2

[a]The errors for the permeability (*P*) and for the selectivity (α) were taken from the range of two measurements.

4.5. Comparison of binary MMMs with literature

Table S9. Comparison of CO_2 permeability and CO_2/CH_4 selectivity for COFs/CTFs as porous filler materials in different polymer MMMs.

Filler	Filler content [wt%]	Matrix	*P* CO_2 [Barrer]	α CO_2/CH_4	Ref.
-	-	Matrimid®	6.8 ± 0.1[a]	30.5± 0.6[a]	[19]
ACOF-1[1]	8	Matrimid®	9.6 ± 1.0[a]	31.9 ± 0.8[a]	
	16		15.3 ± 0.7[a]	32.4 ± 1.8[a]	
-	-	PIM-1[3]	3672[b]	10.6[b]	[20]
SNW-1[2]	10	PIM-1[3]	7553[b]	13.5[b]	
-	-	Pebax® 1657	53[c]	17[c]	[21]
CTPP[4]	0.025	Pebax® 1657	73[c]	25[c]	
-	-	PIM-1[3]	5800[d]	11.5[d]	[22]
FCTF-1	2	PIM-1[3]	7300[d]	16.6[d]	
	5		9400[d]	14.8[d]	
-	-	Matrimid®	6.8 ± 0.3[e]	42 ± 1[e]	This work
CTF-biphenyl	8	Matrimid®	12.0 ± 0.2[e]	43 ± 1[e]	
	16		15.1 ± 0.2[e]	44 ± 1[e]	
	24		15.4 ± 0.5[e]	44 ± 1[e]	
-	-	PSF[5]	5.4 ± 0.0[e]	28 ± 2[e]	[4]
CTF-fluorene	8	PSF[5]	6.6 ± 0.3[e]	32 ± 2[e]	
	16		8.8 ± 0.3[e]	29 ± 0[e]	
	24		12.8 ± 0.1[e]	30 ± 3[e]	
-	-	Matrimid®	6.8 ± 0.3[e]	42 ± 1[e]	
CTF-fluorene	8	Matrimid®	9.2 ± 0.4[e]	43 ± 1[e]	
	16		12.6 ± 0.1[e]	45 ± 1[e]	
	24		17.8 ± 0.3[e]	44 ± 2[e]	
-	-	PSF[5]	7.3 ± 0.2[f]	21 ± 3[f]	[23]
CTF-1	8	PSF[5]	9.2 ± 0.6[f]	21 ± 3[f]	
	16		10.7 ± 0.6[f]	21 ± 3[f]	
	24		12.7 ± 0.8[f]	22 ± 3[f]	

[a]Mixed gas; 308 K; feed pressure 4 bar; [b]Single gas; 303 K; feed pressure 2 bar; [c]Single gas; 293 K; feed pressure 4 bar; [d]Single gas; 303 K; feed pressure 1 atm; [e]Mixed gas; 298 K; feed pressure 4 bar; [f]Single gas; 298 K; feed pressure 3 bar; [1]azine-linked covalent organic framework; [2]Schiff base network; [3]polymer of intrinsic microporosity; [4]porous covalent triazine piperazine polymer; [5]polysulfone

References

1. Bi, J.; Fang, W.; Li, L.; Wang, J.; Liang, S.; He, Y.; Liu, M.; Wu, L. Covalent Triazine-Based Frameworks as Visible Light Photocatalysts for the Splitting of Water. *Macromol. Rapid Commun.* **2015**, *36*, 1799-1805.

2. Bhunia, A.; Boldog, I.; Möller, A.; Janiak, C. Highly stable nanoporous covalent triazine-based frameworks with an adamantane core for carbon dioxide sorption and separation. *J. Mater. Chem. A* **2013**, *1*, 14990.

3. Osadchii, D. Y.; Olivos-Suarez, A. I.; Bavykina, A. V.; Gascon, J. Revisiting Nitrogen Species in Covalent Triazine Frameworks. *Langmuir* **2017**, *33*, 14278-14285.

4. Bügel, S.; Spieß, A.; Janiak, C. Covalent triazine framework CTF-fluorene as porous filler material in mixed matrix membranes for CO_2/CH_4 separation. *Micropor. Mesopor. Mat.* **2021**, *316*, 110941

5. Scholl, R.; Mansfeld, J. meso-Benzdianthron (Helianthron), meso-Naphthodianthron, und ein neuer Weg zum Flavanthren. *Ber. Dtsch. Chem. Ges.* **1910**, *43*, 1734-1746.

6. Kovacic, P.; Jones, M.B. Dehydro Coupling of Aromatic Nuclei by Catalyst-Oxidant Systems: Poly(p-phenylene). *Chem. Rev.* **1987**, *87*, 357-379 357.

7. Dey, S.; Bhunia, A.; Esquivel, D.; Janiak, C. Covalent triazine-based frameworks (CTFs) from triptycene and fluorene motifs for CO_2 adsorption. *J. Mater. Chem. A* **2016**, *4*, 6259-6263.

8. Dey, S.; Bhunia, A.; Boldog, I.; Janiak, C. A mixed-linker approach towards improving covalent triazine-based frameworks for CO_2 capture and separation. *Micropor. Mesopor. Mat.* **2017**, *241*, 303-315.

9. Dey, S.; Bhunia, A.; Breitzke, H.; Groszewicz, P.B.; Buntkowsky, G.; Janiak, C. Two linkers are better than one: enhancing CO_2 capture and separation with porous covalent triazine-based frameworks from mixed nitrile linkers. *J. Mater. Chem. A* **2017**, *5*, 3609-3620.

10. Kuhn, P.; Antonietti, M.; Thomas, A. Porous, Covalent Triazine-Based Frameworks Prepared by Ionothermal Synthesis. *Angew. Chem. Int. Ed.* **2008**, *47*, 3450-3453.

11. Meier, C. B.; Sprick, R. S.; Monti, A.; Guiglion, P.; Lee, J.-S. M.; Zwijnenburg, M. A.; Cooper, A. I. Structure-property relationships for covalent triazine-based frameworks:

The effect of spacer length on photocatalytic hydrogen evolution from water. *Polymer* **2017**, *126*, 283-290.

12. Lim, H.; Cha, M. C.; Chang, J. Y. Preparation of Microporous Polymers Based on 1,3,5-Triazine Units Showing High CO_2 Adsorption Capacity. *Macromol. Chem. Phys.* **2012**, *213*, 1385-1390.

13. Aupoix, A.; Pégot, B.; Vo-Thanh, G. Synthesis of imidazolium and pyridinium-based ionic liquids and application of 1-alkyl-3-methylimidazolium salts as pre-catalysts for the benzoin condensation using solvent-free and microwave activation. *Tetrahedron* **2010**, *66*, 1352-1356.
14. Zhao, R.; Wu, H.; Yang, L.; Ren, Y.; Liu, Y.; Qu, Z.; Wu, Y.; Cao, L.; Chen, Z.; Jiang, Z. Modification of covalent organic frameworks with dual functions ionic liquids for membrane-based biogas upgrading. *J. Membr. Sci.* **2020**, *600*, 117841.
15. Li, H.; Tuo, L.; Yang, K.; Jeong, H.-K.; Dai, Y.; He, G.; Zhao, W. Simultaneous enhancement of mechanical properties and CO_2 selectivity of ZIF-8 mixed matrix membranes: Interfacial toughening effect of ionic liquid. *J. Membr. Sci.* **2016**, *511*, 130-142.
16. Perkin, S.; Crowhurst, L.; Niedermeyer, H.; Welton, T.; Smith, A. M.; Gosvami, N. N. Self-assembly in the electrical double layer of ionic liquids. *Chem. Commun.* **2011**, *47*, 6572-6574.
17. Shindo, R.; Kishida, M.; Sawa, H.; Kidesaki, T.; Sato, S.; Kanehashi, S.; Nagai, K. Characterization and gas permeation properties of polyimide/ZSM-5 zeolite composite membranes containing ionic liquid. *J. Membr. Sci.* **2014**, *454*, 330-338.
18. Kanehashi, S.; Kishida, M.; Kidesaki, T.; Shindo, R.; Sato, S.; Miyakoshi, T.; Nagai, K. CO_2 separation properties of a glassy aromatic polyimide composite membranes containing high-content 1-butyl-3-methylimidazolium bis(trifluoromethylsulfonyl)imide ionic liquid. *J. Membr. Sci.* **2013**, *430*, 211-222.
19. Shan, M.; Seoane, B.; Rozhko, E.; Dikhtiarenko, A.; Clet, G.; Kapteijn, F.; Gascon, J. Azine-Linked Covalent Organic Framework (COF)-Based Mixed-Matrix Membranes for CO_2/CH_4 Separation. *Chem. Eur. J.* **2016**, *22*, 14467-14470.
20. Wu, X.; Tian, Z.; Wang, S.; Peng, D.; Yang, L.; Wu, Y.; Xin, Q.; Wu, H.; Jiang, Z. Mixed matrix membranes comprising polymers of intrinsic microporosity and covalent organic framework for gas separation. *J. Membr. Sci.* **2017,** *528*, 273-283.

21. Thankamony, R.L.; Li, X.; Das, S.K.; Ostwal, M.M.; Lai, Z. Porous covalent triazine piperazine polymer (CTPP)/PEBAX mixed matrix membranes for CO_2/N_2 and CO_2/CH_4 separations. *J. Membr. Sci.* **2019**, *591*, 117348.
22. Jiang, H.; Zhang, J.; Huang, T.; Xue, J.; Ren, Y.; Guo, Z.; Wang, H.; Yang, L.; Yin, Y.; Jiang, Z.; Guiver, M.D. Mixed-Matrix Membranes with Covalent Triazine Framework Fillers in Polymers of Intrinsic Microporosity for CO_2 Separations. *Ind. Eng. Chem. Res.* **2019**, *59*, 5296-5306.
23. Dey, S.; Bügel, S.; Sorribas, S.; Nuhnen, A.; Bhunia, A.; Coronas, J.; Janiak, C. Synthesis and Characterization of Covalent Triazine Framework CTF-1@Polysulfone Mixed Matrix Membranes and Their Gas Separation Studies. *Front. Chem.* **2019**, *7*, 693.
